# Development of a prognostic tool: based on risk factors for tooth loss after active periodontal therapy

**DOI:** 10.1007/s00784-021-04060-x

**Published:** 2021-08-25

**Authors:** Sonja Rahim-Wöstefeld, Dorothea Kronsteiner, Shirin ElSayed, Nihad ElSayed, Peter Eickholz, Bernadette Pretzl

**Affiliations:** 1 Section of Periodontology, Department of Conservative Dentistry, Clinic for Oral, Dental and Maxillofacial Diseases, University Hospital Heidelberg, 69120 Heidelberg, Germany; 2 Private Practice, 68159 Mannheim, Germany; 3 Institute of Medical Biometry and Informatics (IMBI), University Hospital Heidelberg, 69120 Heidelberg, Germany; 4 Department of Periodontology, Center of Dentistry and Oral Medicine (Carolinum), Johann Wolfgang Goethe-University Frankfurt/Main, 60596 Frankfurt, Germany

**Keywords:** Prognostic tool, Model, Periodontal treatment, Tooth loss, Risk factors

## Abstract

**Objectives:**

The aim of this study was to develop a prognostic tool to estimate long-term tooth retention in periodontitis patients at the beginning of active periodontal therapy (APT).

**Material and methods:**

Tooth-related factors (type, location, bone loss (BL), infrabony defects, furcation involvement (FI), abutment status), and patient-related factors (age, gender, smoking, diabetes, plaque control record) were investigated in patients who had completed APT 10 years before. Descriptive analysis was performed, and a generalized linear-mixed model-tree was used to identify predictors for the main outcome variable tooth loss. To evaluate goodness-of-fit, the area under the curve (AUC) was calculated using cross-validation. A bootstrap approach was used to robustly identify risk factors while avoiding overfitting.

**Results:**

Only a small percentage of teeth was lost during 10 years of supportive periodontal therapy (SPT; 0.15/year/patient). The risk factors abutment function, diabetes, and the risk indicator BL, FI, and age (≤ 61 vs. > 61) were identified to predict tooth loss. The prediction model reached an AUC of 0.77.

**Conclusion:**

This quantitative prognostic model supports data-driven decision-making while establishing a treatment plan in periodontitis patients. In light of this, the presented prognostic tool may be of supporting value.

**Clinical relevance:**

In daily clinical practice, a quantitative prognostic tool may support dentists with data-based decision-making. However, it should be stressed that treatment planning is strongly associated with the patient’s wishes and adherence. The tool described here may support establishment of an individual treatment plan for periodontally compromised patients.

## Introduction

Tooth retention remains a serious challenge in periodontally compromised patients. Periodontitis patients have a higher risk of tooth loss when compared to patients without periodontitis [1]. Progressing attachment- and tooth loss pose esthetic and functional limitations and may impair patient’s quality of life and self-confidence [2, 3]. The aim of periodontal therapy is to preserve teeth as long as possible.

There is evidence that some periodontal patients suffer from greater severity early in life or experience a higher risk for disease progression. These patients require specific and individualized treatment planning [4]. Although variables such as bone loss (BL), furcation involvement (FI), probing pocket depth (PPD), number of teeth lost (due to periodontitis), and patient-related factors such as smoking, age, and diabetes affect tooth retention [5–8], it still remains a challenge for the clinician to estimate treatment outcomes. Several attempts have been made to establish a prognostic model [9–13]. However, these models were not based on statistical methodology, and considered mostly tooth-related variables. Current scientific consensus shows that periodontitis represents a multifactorial disease influenced by many risk factors, which individually impinge upon disease progression and treatment management [5, 8, 9, 14–17]. In addition to tooth-related factors, patient-related parameters are strongly associated with tooth loss and need to be taken into account [2, 4].

To date, only a few data-driven prognostic models to estimate long-term tooth retention have been developed [18–20]. The aim of this explorative analysis is to develop a data-based prognostic tool that estimates risk factors contributing to tooth loss in periodontally compromised patients and can be applied in the beginning of the active periodontal therapy (APT).

## Material and methods

A long-term project was initiated in 2002 at the university hospital in Heidelberg to analyze tooth-retention in patients after comprehensive periodontal treatment. Detailed descriptions of the study protocol have been published [5, 6, 8, 17]. Thus, we only give a brief description of the methodologic aspects of the study.

Patients, who had received periodontal therapy (anti-infective therapy with subgingival debridement and periodontal surgery, if required) between 1992 and 1997 at the Section of Periodontology, Department of Conservative Dentistry under the lead of one periodontal specialist (PE), were re-examined 10 years ± 6 months after initiation of APT (T2) by an independent periodontal specialist (BP) [5, 6].

### Radiographic examination

Every patient had obtained a complete set of periapical radiographs at the beginning of APT. A Schei-ruler was used to evaluate interproximal bone loss [21]. Teeth were assigned to one of five increments of BL (≤ 20%; 21% to < 40%; 40% to < 60%; 60% to < 80%; ≥ 80%; Pretzl et al., 2008) according to BL at the most affected site. These thresholds correspond roughly to the staging of periodontitis in the current classification with BL ≤ 20% representing stage I and BL 21% to < 40% approximately stage II. If a tooth experienced vertical bone loss, it was assigned to one of three groups: shallow (< 2 mm), moderate (2.5–4 mm), or deep (≥ 4 mm).

### Evaluation of patients’ charts

To assess tooth loss, patients’ charts were analyzed, and tooth number at baseline (T0), first SPT examination (T1), and 10-year re-evaluation (T2) was compared.

Additionally, the following variables were retrieved from patient’s medical history at baseline (T0): age, smoking status (current/former (quit smoking at least 5 years ago)/never smoker) [22], and self-reported diabetes (yes/no).

PCR-value [23] at the beginning of supportive periodontal therapy (SPT, T1) was included in this analysis.

Further tooth-related parameters were retrieved from patients’ charts (T0): localization (maxilla/mandible), tooth type (anterior/pre-molar/molar), FI (single-rooted tooth/multi-rooted tooth with/without furcation involvement), and abutment tooth (none/abutment tooth for fixed/removable prosthodontic construction).

### Supportive periodontal therapy

During SPT, a standardized protocol including oral hygiene instructions and supragingival plaque removal was performed. Dental and periodontal status was obtained once to twice a year. If PPDs were 4 mm with BOP or ≥ 5 mm, subgingival scaling was performed [24]. If subgingival debridement of more than five teeth was necessary, complete re-treatment was recommended. However, only a limited number of patients received re-treatment during SPT, which complied with a non-surgical approach. Since October 1999 patients have been assigned an individual SPT interval according to the periodontal risk assessment (PRA) [5, 22].

### Statistical analysis

Descriptive methods were used to summarize characteristics on patient- and tooth-level. Continuous variables were documented using mean and standard deviation, categorical variables using absolute and relative frequencies.

Third molars were not included in the analysis.

Data entry was performed using Microsoft Excel^©^ for macOS (Version 16.29.1; Microsoft Corporation, Redmond, WA, USA). One investigator (SR-W) entered all data into one file. An independent statistician (DK) calculated the descriptive analysis and modeled the generalized linear-mixed model-tree using R version 4.0.2 [25].

### Development of prognostic model

A generalized linear mixed model tree was used to develop a prognostic model for tooth loss on the basis of periodontally treated patients 10 years after APT. The generalized linear mixed model tree performs automated variable selection and allows considering the nested structure of tooth within a patient [26]. The independent variables taken into consideration were age (years), gender (female/male), PCR, smoking (active/former/never), diabetes (yes/no) on patient-level, as well as jaw (maxilla/mandible), tooth type (anterior/pre-molar/molar), FI (single-rooted/multi-rooted without/with furcation involvement), interproximal BL (≤ 20%/21% to 40%/41% to 60%/61% to 80%/ > 80%), infrabony defect (shallow/moderate/deep), and abutment tooth (no/fixed/removable dentures) on tooth-level. Because of the low counts in interproximal bone loss categories 4 and 5, these groups were merged (interproximal bone loss > 60%).

The present study evaluates primary tooth-related risk factors for tooth loss. For all analyses, the basic level tooth was clustered into the upper level patient; the patient was considered as random effect.

In terms of risk factor selection, 200 bootstrap samples of the data were used to achieve robust variable selection results. Bootstrapping is done on patient level meaning that patients are randomly drawn with replacement such that the bootstrap data includes the same number of patients as the original data set. For each bootstrap sample, a generalized linear mixed model tree is fitted, and the occurrence of the selected variables was counted. Variables on tooth-level selected by more than 50% and on patient-level by more than 25% of the bootstrap-based models were taken into consideration for the selection within the final model, because bootstrap samples are based on patients, which limit the variability in patient level characteristics. To build the final tree, the model was fitted on all samples using the variable set selected by applying the bootstrap approach.

After the set of variables was fixed, the prognostic model was internally validated in order to avoid overfitting. Ten-fold cross-validation (CV) was used to estimate the area under the receiver operating characteristic curve (AUC), sensitivity, and specificity for evaluating the model fit. Cross-validation means splitting the data in 10 parts, 9 splits are used for training the model, and each split is used for testing the model once. Cross-validation sets were split by patient, which means all teeth of a patient were included in the same fold to keep training and test set independent. Consequently, CV folds included the same number of patients, but may not include the same number of teeth, as the number of teeth differs between patients. The mean value of AUC, sensitivity, and specificity was calculated over the cross-validation sets. They were reported together with 95% Wilson confidence intervals (CI) based on the number of independent patients, which reflects a conservative approach. The generalized linear mixed model tree generates a probability for tooth loss after 10 years for each tooth individually.

## Results

Patient- and tooth-related characteristics of the sample upon which the model is based can be found in Tables 1 and 2.

**Table 1:** Descriptive statistics and univariate analysis of patient characteristics

Variable	Total number (%)	Mean (SD)
Number of patients	110	
Gender		
Female	68 (61.8%)	
Male	42 (38.2%)	
Diabetes		
Non-diabetic	101 (91.8%)	
Diabetic	9 (8.2%)	
Smoking		
Non-smoker	50 (45.5%)	
Former smoker (quit smoking at least 5 years ago)	27 (24.5%)	
Active smoker	33 (30.0%)	
Smoking		
Non-smoker (non-smoker + former smoker)	77 (70.0%)	
Active smoker	33 (30.0%)	
Age	110	46,7 (10.26)
Plaque Control Record (T1)	103	31.3 (17.39)

**Table 2:** Descriptive statistics and univariate analysis of tooth characteristics

Variable	Total number	Tooth retention		Tooth loss	*p* value
Number of teeth — after 10 years	2556	2390		166	
Jaw					0.001
Maxilla	1215	1119 (92.1%)		96 (7.90%)	
Mandible	1341	1271 (94.78%)		70 (5.22%)	
Tooth type					< 0.001
Anterior	1207	1160 (96.11%)		47 (3.89%)	
Pre-molar	733	682 (93.04%)		51 (6.96%)	
Molar	616	548 (88.96%)		68 (11.04%)	
Furcation (missing n = 14)	•				< 0.001
Single-rooted teeth	1770	1690 (95.48%)		80 (4.52%)	
Multi-rooted teeth without FI	341	312 (91.50%)		29 (8.50%)	
Multi-rooted teeth with FI	431	378 (87.30%)		53 (12.30%)	
Abutment tooth (missing n = 14)	•				< 0.001
No abutment tooth	2243	2119 (94.47%)		124 (5.53%)	
Abutment tooth — fixed	232	207 (89.22%)		25 (10.78%)	
Abutment tooth — removable	67	54 (80.60%)		13 (19.40%)	
Bone loss (missing n = 28)					< 0.001
Periodontal bone loss ≤ 20%	700		679 (97.0%)	21 (3.00%)	
Periodontal bone loss 21–40%	1078		1034 (95.92%)	44 (4.08%)	
Periodontal bone loss 41–60%	531		484 (91.15%)	47 (8.85%)	
Periodontal bone loss 61–80%	160		131 (81.87%)	29 (18.13%)	
Periodontal bone loss > 80%	59		39 (66.10%)	20 (33.90%)	
Vertical bone loss (missing n = 28)					0.001
Shallow (2 mm)	2260		2130 (94.25%)	130 (5.75%)	
Moderate (2.5–4 mm)	266		235 (88.35%)	31 (11.65%)	
Deep (≥ 4 mm)	2		2 (100.00%)	0 (0.00%)	

*P* values of the Chi-squared test are presented for completeness but are not considering the clustered structure of the data and have thus to be interpreted with care.

In the sample 61.8% of patients were female, average age at T0 was 46.7 years (standard deviation (SD) = 10.26) and 30.0% were active, 24.5% former, and 45.5% non-smokers. Nine participants suffered from diabetes mellitus (8.2%). The average PCR at T1 was 31.3% (SD = 17.39).

At T0 2556 teeth were present. Of these 1341 teeth were in the mandible, 1215 in the maxilla, 733 teeth were pre-molars, 616 molars, and 1207 anteriors. More than two thirds were single-rooted (1770) and 431 teeth exhibited a FI. The majority of teeth had an interproximal BL of 21–40% (1078), while 160 teeth showed an interproximal BL between 61 and 80%, and 59 the most severe BL (> 80%). A total of 232 teeth were abutment teeth used for fixed (10.3%) and 67 for removable prosthetic reconstructions (2.9%). One hundred sixty-six teeth were lost during ten years of SPT (0.15/year/patient). Table 2 shows the distribution of tooth loss.

### Prognostic tree model

**Fig. 1:**
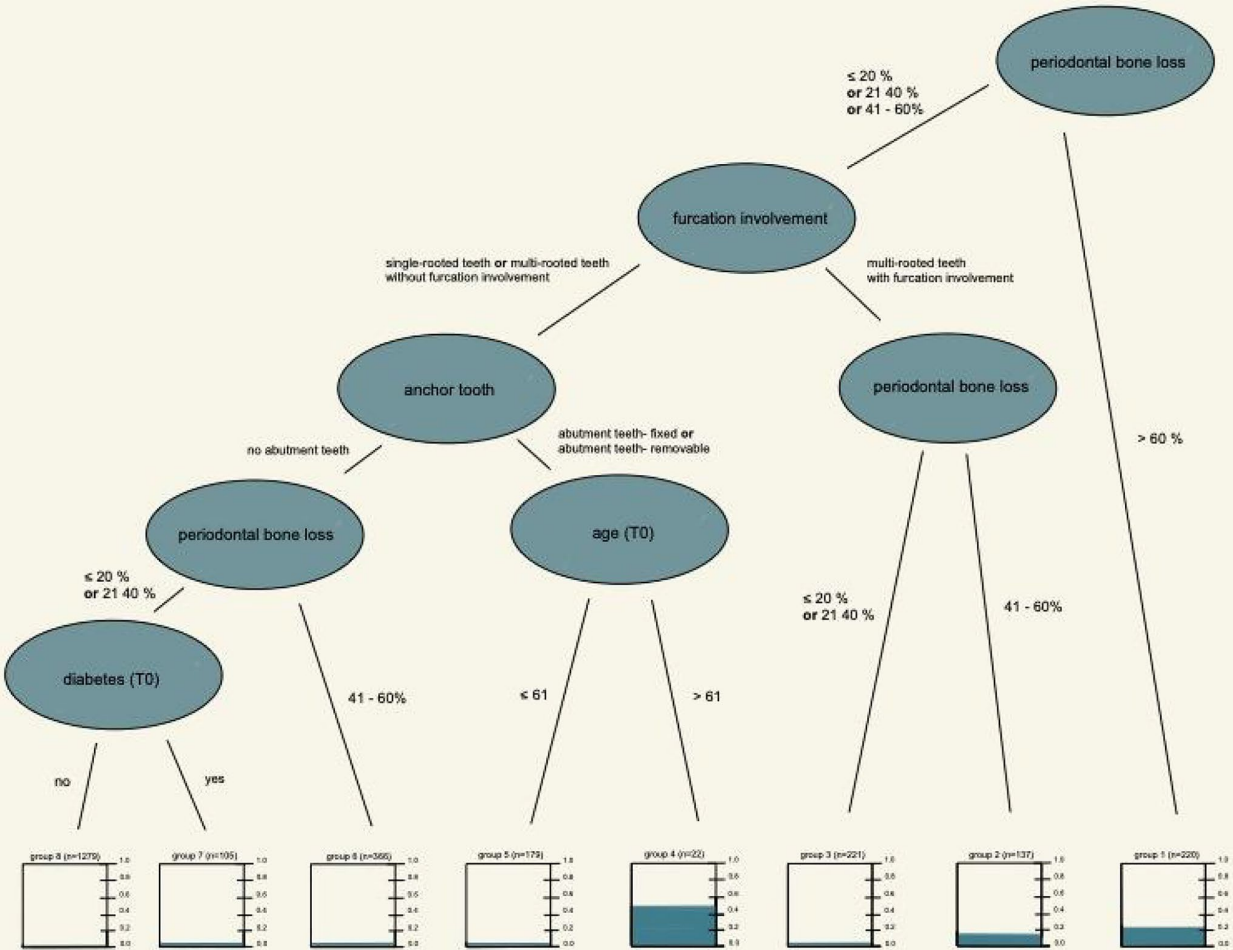
Generalized linear mixed model tree

For the final tree model, data of 110 patients with 2528 teeth were used corresponding to a complete case analysis. The final tree model, where variable selection was automatically performed based on a bootstrap approach including patient- and tooth-related variables: BL, FI, abutment tooth, age (≤ 61 versus > 61), and diabetes (Table 3 and Fig. 1).

**Table 3:** Generalized linear mixed model tree — groups

Group number	Group	Rate of tooth loss after 10 years (teeth lost/total number of teeth)
8	Periodontal bone loss > 60%	22.3% (49/220)
7	Periodontal bone loss 41–60% & Multi-rooted teeth with furcation involvement	16.8% (23/137)
6	Periodontal bone loss ≤ 40% & Multi-rooted teeth with furcation involvement	6.3% (14/221)
5	Periodontal bone loss ≤ 60% & Single-rooted teeth or multi-rooted teeth Without furcation involvement & Abutment tooth — fixed or abutment tooth — removable & Age > 61 years	50.0% (11/22)
4	Periodontal bone loss ≤ 60% & Single-rooted teeth or multi-rooted teeth Without furcation involvement & Abutment tooth — fixed or abutment tooth — removable & Age ≤ 61 years	5.0% (9/179)
3	Periodontal bone loss 41–60% & Single-rooted teeth or multi-rooted teeth Without furcation involvement & No abutment tooth	5.7% (21/366)
2	Periodontal bone loss ≤ 40% & Single-rooted teeth or multi-rooted teeth Without furcation involvement & No abutment tooth & Diabetic	5.7% (6/105)
1	Periodontal bone loss ≤ 40% & Single-rooted teeth or multi-rooted teeth Without furcation involvement & No abutment tooth & No diabetic	2.2% (28/1279)

Based on a statistical model, the tree selected the variable with the greatest impact on tooth loss in the first step. In the present analysis, periodontal BL over 60% was marked as first cut-off. Teeth in this category are at risk of being lost in 22.3%. In further steps, additional variables were chosen accordingly. In the end, teeth were split into eight categories/groups (Table 3). Teeth with a periodontal BL 41–60% and being a multi-rooted tooth with FI were lost in 16.8%, whereas teeth with a periodontal BL < 40% and being multi-rooted with FI were lost in 6.3%. In category 5 and 4 age seems to have a great impact. While teeth with a periodontal BL ≤ 60% and being single-rooted or multi-rooted without FI and have an abutment function for fixed or removable dentures were lost in only 5.0% of the cases in patients ≤ 61 years of age, patients over the age bracket of 61 years in the same category were at risk of losing these teeth in 50.0%. Teeth with an initial BL of 41–60% and being single-rooted or multi-rooted without FI and have no abutment function were at risk of being lost in 5.7%. When teeth experience a periodontal BL of < 40% and being single-rooted or multi-rooted without FI and have no abutment function and the patient suffered from diabetes, teeth had a risk of being lost in 5.7%. However, when the patient did not suffer from diabetes, teeth were only lost in 2.2% of the cases. Note that a higher category number does not correspond to higher tooth loss rate. Rates for tooth loss within each category are given in Table 3.

To evaluate the performance of the fitted model (internal validation) CV-folds were used. They reached a mean AUC-value of 0.77 (95% CI [0.68; 0.84]), a sensitivity of 0.73 (95% CI [0.64; 0.80]), and a specificity of 0.79 (95% CI [0.70; 0.86]). These values can be considered as acceptable model performance. In this setting, a higher specificity is desirable corresponding to the probability of teeth correctly classified as “no tooth loss.” The naïve model performance, i.e., evaluation measures calculated on the trainings set, reached an AUC-value of 0.95, a sensitivity of 0.80, and a specificity of 0.93. It is important to note that these naïve measures overfit the model performance and might not be reproducible in an external cohort.

## Discussion

Treatment of periodontally compromised patients remains a challenge. Rather than the therapy itself, treatment planning represents a critical and important part.

The presented prognostic tool aims to support practitioners’ decision-making, while establishing a treatment plan in the beginning of periodontal therapy. To evaluate individual tooth prognosis, factors on different levels have to be taken into account: tooth, patient, dentist, and environmental level [4–6, 17, 27–32]. The final tree model evaluates tooth- and patient-related variables and estimates the risk for tooth loss based on a statistical model. Abutment teeth (fixed or removable) with a periodontal BL ≤ 60%, being single-rooted or multi-rooted without FI in patients > 61 years of age are considered to be at highest risk for tooth loss (50.0%). In comparison, the risk of tooth loss in teeth with the same criteria, but under the age of 61, is estimated to be ten times less (5.0%) (Table 3). Thus, increasing age plays an important role in our evaluation. This finding corresponds with previous findings from other surveys, where age was considered a risk indicator for periodontal disease progression [16, 33]. An accumulation of plaque, biological changes, and consequently a reduction of tissue regeneration and disease regression may lead to an impaired wound-healing ability with increasing age [7, 34–37].

In the past, surveys presented prognostic systems to evaluate the risk for tooth loss [9–13]. However, most of these publications did not introduce an evaluated data-driven prognostic model to avoid overfitting. A recent publication introduced a nomogram to predict periodontal tooth loss based on the staging and grading system [38], but tooth loss was modeled on patient-level only.

Hirschfeld and Wasserman categorized teeth into “favorable and questionable” prognoses—based on tooth-related factors such as FI, PPD, alveolar BL, and degrees of mobility [9]. Checchi et al. (2002) distinguished “good/questionable/or hopeless” teeth based on tooth-related factors [10]. However, the assignment of prognosis in these two studies relies on p values, without focusing on model performance. Both studies conclude the highest risk for loss to be in teeth with severe periodontal BL (> 75%). Meanwhile, several studies have shown that teeth with such BL can be retained over a long period (10 years and more), implicating that both of these systems do not seem suitable for treatment planning [5, 17, 39–42]. The present prognostic tool appears to show a higher degree of differentiation, since teeth with advanced BL of 60% seem to have a low risk of tooth loss (23%; Table 3, Fig. 1), and teeth with BL < 60% without FI, used as abutment teeth in patients aged > 61, have a higher risk of being lost (50%, group 4, Table 3, Fig. 1). Additionally, patient-related factors are disregarded in the two aforementioned studies. All variables have to be interpreted within the patient, since teeth belong to a person, whose characteristics or behavior influence the outcome as well [17].

A report by Kwok and Caton (2007) considered general (compliance, plaque accumulation, smoking, diabetes, and others) and local factors (PPD, attachment loss, anatomic plaque-retentive factors, trauma from occlusion and habits, mobility) based on literature evidence. No statistical model was calculated based on clinical data, and the relative weight of each variable was not presented, which results in a prognostic proposal with uncertain recommendations [11]. A direct comparison between their narrative review and our statistical data-driven model-tree does therefore not seem feasible.

Nibali et al. proposed a different prognostic system [13]: teeth were assigned either a good, fair, questionable, or unfavorable prognosis based on a periodontal risk assessment (PRA [22]) and periodontal risk calculator (PRC [43]). Variables (such as PPD, FI, mobility, BL, periapical pathology, restorability) were taken into account from previous literature. Although their statistical methodology considers the clustered structure of the data, the prognostic system was based on risk assessment in patients after APT. In contrast to this, the prognostic tool described in this study attempts to assess the risk for tooth loss in periodontally compromised patients at the beginning of treatment in order to support treatment planning. Furthermore, the two periodontal risk assessment methods (PRA and PRC) do not seem to have a high level of agreement in terms of patient’s individual risk for disease progression [44].

Mc Gowan et al. proposed an evidence-based prognostic model in their review using previously published periodontal prognosis models [12]. Variables included tooth-level (BL/age-ratio, PPD, extent of FI, infrabony defect, compromising anatomical factors, extent of mobility) and patient-level (smoking, poorly controlled diabetes, BOP). However, they present a comparison of models and do not provide an original tool.

Only a few groups have developed a data-based prognostic model to estimate long-term tooth retention [18–20]. Faggion et al. (2017) developed a prognostic model to estimate survival rates of teeth in periodontally compromised patients over an observation period of 11.8 years. In agreement with our findings, variables such as diabetes and BL (additionally tooth mobility and root type) were identified as influential predictors for tooth loss. Their prognostic model showed that multi-rooted, vital teeth in non-diabetic patients with a periodontal bone loss of 40% have a probability of 80–89% tooth survival. In contrast patients suffering from diabetes in the same group showed a 50–59% probability of tooth survival. These results are comparable to ours (Table 3). Diabetes patients with single-rooted or multi-rooted teeth without furcation involvement and no abutment function with a periodontal bone loss ≤ 40% showed a 5.7% rate of being lost. On the other hand, the rate in patients without diabetes with the same criteria was calculated at 2.2% (Fig. 2). However, variables in Faggion et al. (2017) were identified using a backward-selection procedure based on p values. P values depend on various circumstances, i.e., higher sample sizes lead to smaller p-values (even for variables which may not help to predict the outcome). In contrast, our statistical approach relies on automatically selecting risk factors and cut-offs.

In contrast to our tool which is applicable for all teeth, Miller et al. proposed a prognostic model for molars [19]. The selection of variables influencing the score (molar type, smoking, FI, PPD, mobility, and age) was not based on statistical methods but quantitatively. This subjective scoring may lead to poor model performance and bias. In comparison, our analysis combines variable selection and outcome prediction and allows for an impartial objective data selection.

The analysis of Martinez-Canut et al. (2018) consists of two separate models (molars and non-molars) analyzing one factor at a time as opposed to our explorative analysis, where all teeth are included simultaneously in one model. Separating teeth in different models leads to lower sample sizes, which may result in less accurate predictions. Additionally, multiple factors interact in one patient and should therefore be considered together in a data-driven tool, as well. In line with the present study, Martinez-Canut et al. calculated discrimination measurements such as AUC, sensitivity, and specificity. In their study, AUC amounts to 0.93–0.97 (molars/non-molars); with a sensitivity of 39%/43%, and a specificity of 98%/99%. The introduced prognostic tool reaches an AUC value of 0.77, a sensitivity of 73%, and a specificity of 79%. The difference may be explained by the fact that the presented analysis uses cross-validation for internal validation. These statistical methods are crucial to avoid overfitting and overoptimistic results but are occasionally used in the evaluation of already existing prediction models for tooth loss.

In Ancient Greece “prognosis” was described by Hippocrates as “foreseeing and foretelling, by the side of the sick, the present, the past, and the future” [45]. The appreciation of past and present helps to better understand the future. This concept still applies today. Treatment outcome is strongly influenced by patient’s history as well as adherence. Studies have reported the significant impact of compliance on tooth loss, emphasizing that patients, who do not adhere to treatment regimen, loose significantly more teeth than others [17, 20, 29, 31]. Unfortunately, factors such as patient adherence and dentist’s skills cannot be predicted or measured in advance of periodontal therapy. Theoretically, patient adherence could be taken into account indirectly using PCR trying to assess a patient’s oral hygiene effectiveness. Although PCR at T1 was recorded, the final tree model did not take this variable into account, since the plaque formation rate can vary widely between patients. Nonetheless, we know from numerous studies that patient adherence and dentists’ preferences play a decisive role in long-term tooth retention. Thus, they have to be taken into account apart from the tool. The same applies to the variable “smoking.” The reason why smoking was not considered may be the small difference between the number of teeth lost in smokers vs. non-smokers and the small sample size, which may have weakened the effect of smoking. Additionally, in many populations as well as in our sample, smoking prevalence decreased due to the increasing awareness campaign and frequent smoking cessation during SPT [46].

Prognosis at start of therapy is only the first step to guide treatment decisions. Prognosis means forecasting the future with the knowledge of the past and the present. This forecast will be accurate as long as past and present conditions stay the same. However, people change to the better (quit smoking) or the worse (develop diabetes or depression). Thus, prognosis at start of treatment will never 100% accurately forecast the course of periodontal disease including tooth loss. In the course of SPT the dentist will try to influence the patient to the better or to adjust treatment to ameliorate the worse. However, the result of this process cannot be exactly predicted at baseline even not by best prognosis tool.

## Perspective

The presented prognostic tool was cross-validated to avoid overfitting. Whether this prognostic model is appropriate to evaluate the risk for tooth loss in other groups has yet to be verified. To review the accuracy and the general applicability, an external validation with a different cohort is already planned.

The setting of the study may represent a limitation. Practice-based studies report lower tooth loss rates [9, 15, 47] compared with university-based studies [7, 18, 41, 48]. Different patients treated in these settings may explain the difference. To verify whether the presented tool is applicable in a practice-based setting, another validation process should be conducted.

## Limitations

The results have to be interpreted with caution. There might be other patient- or tooth-related characteristics that influence tooth survival not observed in the present study.

The tool presented here relies on mathematical models allocating relative weight to the presented variables. It does not differentiate in different categories (good/questionable/hopeless) or scores, because such cutoffs result in a loss of information and are difficult to choose. Therefore, the probability of tooth loss rather than a category is reported.

## Conclusions

In clinical practice, a quantitative prognostic tool may support dentists with data-based decision-making and enable an individual treatment plan for periodontally compromised patients. However, it should be stressed that treatment planning is strongly associated with patient’s wishes and adherence. In light of this, the presented prognostic tool may be of supporting value.
